# Supplementary material for: Fleshy red algae mats act as temporary reservoirs for sessile invertebrate biodiversity
Source: Commun Biol. 2022 Jun 13;5:579. doi: 10.1038/s42003-022-03523-5 (PMC9192683; doi:10.1038/s42003-022-03523-5)
Supplement: Supplementary file 2 — Supplementary Material [file 42003_2022_3523_MOESM2_ESM.pdf]

Supplementary Material (SM) to:

Fleshy red algae mats act as temporary reservoirs for sessile invertebrate biodiversity

Yusuf C. El-Khaled[1]*, Nauras Daraghmeh[1,2], Arjen Tilstra[1], Florian Roth[3,4], Markus Huettel[5], Felix I. Rossbach[1], Edoardo Casoli[6], Anna Koester[1], Milan Beck[1], Raïssa Meyer[1], Julia Plewka[1], Neele Schmidt[1], Lisa Winkelgrund[1], Benedikt Merk[1], Christian Wild[1]

SM 1 – Detailed table highlighting the number of phenotypes and individuals per taxon in total and per m^2 habitat (*P. crispa*, *P. oceanica* leaves, rhizome and holobiont)

Table S1: Mean numbers of individuals/colonies (Ind.) and phenotypes (PT) per taxon and per m^2 habitat identified in *Phyllophora crispa*, *Posidonia oceanica* holobiont, *P. oceanica* leaves and *P. oceanica* rhizome.

	P. crispa		*P. oceanica* holobiont		*P. oceanica* leaves		*P. oceanica* rhizome	
	PT	Ind.	PT	Ind.	PT	Ind.	PT	Indi.
Ascidiacea	9	223.03 ± 54.00	7	178.82 ± 45.04	4	197.16 ± 68.54	4	152.24 ± 47.32
Bryozoa	76	44222.25 ± 5474.96	78	7654.80 ± 681.14	48	6013.22 ± 650.41	50	10035.09 ± 1190.28
Cnidaria	4	24.93±10.59	6	122.91 ± 42.38	4	156.17 ± 67.43	2	74.67 ± 31.98
Entoprocta	1	425.65 ± 353.20	0	0	0	0	0	0
Foraminifera	81	11145.08 ± 900.71	52	7610.81 ± 775.65	33	7316.31 ± 1101.70	31	8037.82 ± 1021.76
Mollusca (Bivalvia)	4	112.07 ± 34.50	4	37.58 ± 26.72	2	12.35 ± 9.58	2	74.15 ± 63.08
Polychaeta (Sedentaria)	23	5949.83 ± 617.91	13	3734.15 ± 578.82	4	2145.46 ± 454.98	11	6037.74 ± 1061.84
Rotifera	1	255.07 ± 45.49	0	0	0	0	0	0
Porifera	24	1649.17 ± 151.50	13	195.81 ± 45.64	2	16.99 ± 9.99	17	455.10 ± 81.31
Total	223	64007.06 ± 5609.48	179	19535.88 ± 1420.98	97	15857.67 ± 1654.42	117	24866.81 ± 1990.69

SM 2 – Detailed results of statistical analysis

Table S 2: Results of Dunn Kruskal-Wallis multiple comparison for calculated abundances of individuals per habitat m^{-2} of *Phyllophora crispa*, *Posidonia oceanica* holobiont, *P. oceanica* leaves and *P. oceanica* rhizome, *p* values presented as either adjusted using the Bonferroni method or unadjusted.

Comparison	Z	*p* adjusted	*p* unadjusted
P. crispa – *P. oceanica* holobiont	-3.568	0.000	0.002
P. crispa – *P. oceanica* leaves	-8.297	0.000	0.000
P. crispa – *P. oceanica* rhizome	6.166	0.000	0.000
P. oceanica holobiont – *P. oceanica* leaves	-6.643	0.000	0.000
P. oceanica holobiont – *P. oceanica* rhizome	3.923	0.000	0.001
P. oceanica leaves – *P. oceanica* rhizome	-1.773	0.076	0.457

Table S 3: Results of permutational analysis of variance (PERMANOVA) with subsequent pair-wise test for sessile invertebrate communities (incidence data) with the factor 'habitat' (i.e., *Phyllophora crispa*, *Posidonia oceanica* leaves, *P. oceanica* rhizome) with Bray Curtis similarity, Type III (partial), unrestricted permutations (perms) of raw data (incidence) and Monte Carlo tests (MC), and the 999 permutations.

Groups	t	*p* (perm)	Unique perms	*p* (MC)
P. crispa – *P. oceanica* leaves	5.0657	0.001	999	0.001
P. crispa – *P. oceanica* rhizome	4.3302	0.001	999	0.001
P. oceanica leaves – *P. oceanica* rhizome	4.9623	0.001	999	0.001

Table S 4*:* Results of Dunn Kruskal-Wallis multiple comparison for calculated abundances of individuals per seafloor m^{-2} (i.e., considering enlargement factor) of *Phyllophora crispa*, *Posidonia oceanica* holobiont, *P. oceanica* leaves and *P. oceanica* rhizome, *p* values presented as either adjusted using the Bonferroni method or unadjusted.

Comparison	Z	*p* adjusted	*p* unadjusted
P. crispa – *P. oceanica* holobiont	-3.788	0.000	0.001
P. crispa – *P. oceanica* leaves	-5.007	0.000	0.000
P. crispa – *P. oceanica* rhizome	7.368	0.000	0.000
P. oceanica holobiont – *P. oceanica* leaves	-2.000	0.045	0.273
P. oceanica holobiont – *P. oceanica* rhizome	5.204	0.000	0.000
P. oceanica leaves – *P. oceanica* rhizome	3.138	0.002	0.010

SM 3 – Detailed information on enlargement factor calculations

Table S 5: Mean Enlargement Factors (EL), respective replication, standard deviations (SD) and standard errors (SE) for *Phyllophora crispa*, *Posidonia oceanica* leaves, *P. oceanica* rhizomes and *P. oceanica* holobiont.

	P. crispa	*P. oceanica* holobiont	*P. oceanica* leaves	*P. oceanica* rhizome
Mean EL	4.87	8.33	7.33	1.99
Replication	64	32	32	32
SD	1.52	2.94	2.89	0.43
SE	0.19	0.52	0.51	0.08

SM 4 – Details of biodiversity assessment calculations

Table S 6: Sample completeness profiles, asymptotic and empirical estimates and resulting undetected diversity, non-asymptotic rarefaction and extrapolation as well as evenness analysis for phenotype richness (q = 0), Shannon diversity (q = 1) and Simpson diversity (q = 2), and Pilou's *J'* for evenness calculated for *Phyllophora crispa*, *Posidonia oceanica* holobiont, *P. oceanica* leaves and *P. oceanica* rhizome.

		***q* = 0**	***q* = 1**	***q* = 2**
Step I: Sample completeness				
P. crispa		85.67 %	95.80 %	99.37 %
P. oceanica holobiont		72.97 %	94.77 %	99.67 %
P. oceanica leaves		79.33 %	95.99 %	99.69 %
P. oceanica rhizome		73.57 %	92.04 %	99.09 %
Step II: Asymptotic and empirical diversity estimates and resulting undetected diversity				
P. crispa				
	Asymptotic	260.31	170.04	132.15
	Empirical	223.00	155.58	126.12
	Undetected	37.31	14.46	6.03
P. oceanica holobiont				
	Asymptotic	245.31	118.27	79.33
	Empirical	179.00	105.44	75.29
	Undetected	66.31	12.83	4.04
P. oceanica leaves				
	Asymptotic	122.28	68.94	49.11
	Empirical	97.00	62.53	46.76
	Undetected	25.28	6.41	2.35
P. oceanica rhizome				
	Asymptotic	159.03	89.53	60.59
	Empirical	117.00	76.69	56.45
	Undetected	42.03	12.84	4.14
Step III: Non-asymptotic coverage-based rarefaction and extrapolation: diversity estimates at C_{max}				
	Maximum standardised coverage C_{max} = 96.9 %			
P. crispa		234.08	158.65	127.28
P. oceanica holobiont		206.63	110.83	76.81
P. oceanica leaves		102.74	63.93	47.25
P. oceanica rhizome		142.81	84.04	58.44
Step IV: Evenness				
	Maximum standardised coverage C_{max} = 96.9 %			
P. crispa		0.93*	0.68	0.54
P. oceanica holobiont		0.88*	0.593	0.37
P. oceanica leaves		0.90*	0.62	0.45
P. oceanica rhizome		0.89*	0.59	0.41

*for Pilou's *J'* instead evenness at q = 0

SM 5 – Map of Giglio Island and Sampling Points

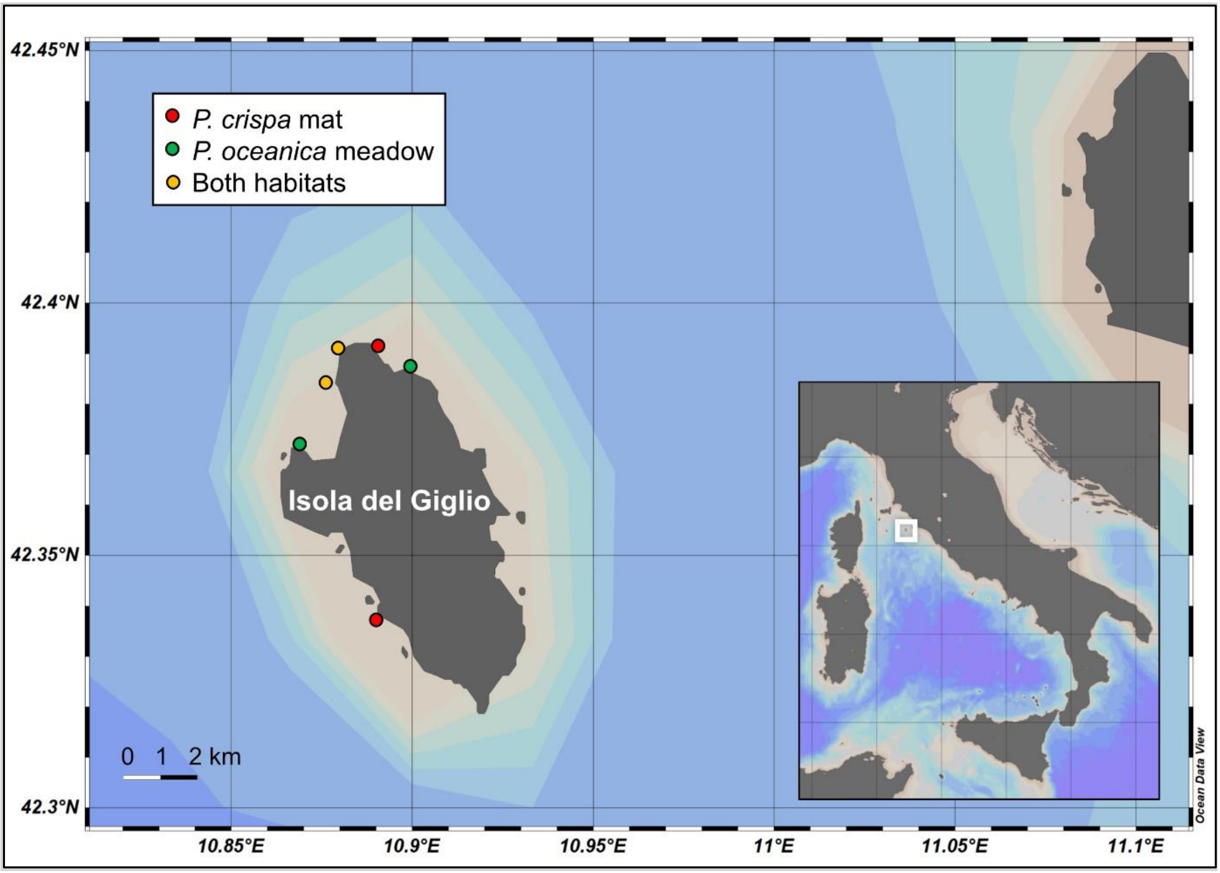

Figure S 1: Location of Giglio Island and sampling sites in the northern part of the Tyrrhenian Sea. Locations (clockwise, starting with the southernmost): "Corvo": 42°20'17.74"N 10°53'22.48"E; "3 Fratelli": 42°22'5.77"N 10°52'14.80"E; "Secca 2": 42°23'1.85"N 10°52'42.12"E; "Fenaio"_ 42°23'19.98"N 10°52'47.92"E; "Punta del morto": 42°23'22.20"N 10°53'21.07"E; "Cala Calbugina": 42°23'6.09"N 10°53'51.24"E; Red dots indicate *P. crispa* sampling sites, green dots indicate *P. oceanica* sampling sites and yellow dots indicate sampling sites where both *P. crispa* and *P. oceanica* were sampled. Number of sampled specimens were 4x "Corvo", 4x "Fenaio", 4x "Punta del Morto", and 4x "Secca 2" for *P. crispa*; 10x "3 Fratelli", 5x "Fenaio", 5x "Cala Calbugina", and 9x "Secca 2" for *P. oceanica* leaves; 10x "3 Fratelli" and 10x "Secca 2" for *P. oceanica* rhizomes. Made with Ocean Data View according to Schlitzer (2016) [1].

<u>SM 6 – Schematic representation of *P. oceanica*</u>

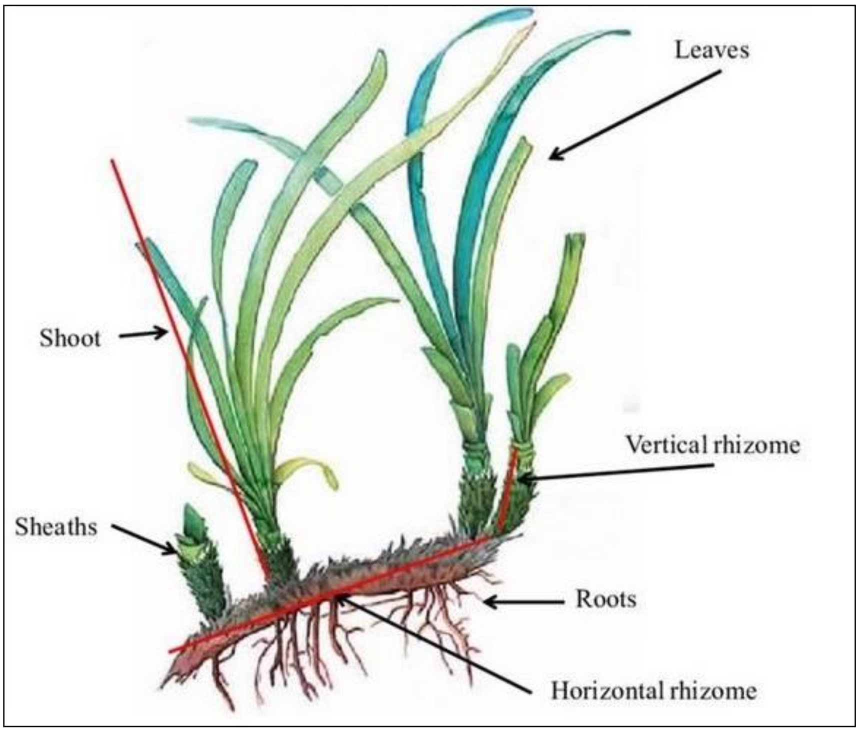

Figure S 2: Schematic overview of *P. oceanica* seagrass. For the present study, leaves were sampled as displayed. “Rhizomes”, as defined in the ‘Material’ section, consisted of the sheaths, both vertical and horizontal rhizome, as well as the upper layers of the roots. Figure taken from Vasapollo (2010) [2].

SM 7 – Literature used for identification of species/phenotypes

Table S 7: Literature used for identification of species/phenotypes in *P. crispa* and *P. oceanica* samples.

**Author(s)**	**Year**	**Title**
Riedl R	2011	Fauna und Flora des Mittelmeeres [3]
Stresemann E	1992	Wirbellose [4]
Milker Y, Schmiedl G	2012	A taxonomic guide to modern benthic shelf foraminifera of the western Mediterranean [5]
Murray JW	1979	British Nearshore Foraminifera [6]
Cimerman F, Langer MR	1991	Mediterranean Foraminifera [7]
Holbourn A, et al.	2013	Atlas of Benthic Foraminifera [8]
Zabala M, Maluguer P	1988	Illustrated keys for the classification of Mediterranean Bryozoa[9]
Hayward JP, Ryland JS	1999	Marine Fauna of North-West-Europe [10]
Bianchi, CN	1981	Guide per il riconoscimento delle specie animali delle acque lagunari e costiere italiane [11]
Ten Hove HA, Kupriyanova AK	2009	Taxonomy of Serpulidae (Annelida, Polychaeta): the state of affairs [12]
Zibrowius H	1968	Etude mophologique, systématique et écologique des Serpulidae (Annelida Polychaeta) de la région de Marseille [13]
Zibrowius H	1972	Mise au point sur les especes mediterraneennes de Serpulidae (Annelida Polychaeta) déecrites par Stefano delle Chiaje (1822-1829, 1841-1844) et Oronzio Gabriele Costa (1861) [14]

SM 8 – Subset dataset "Polychaeta"

Table S 8: Observed Polychaeta phenotypes that were identified to species (green), genus (orange), family (blue), order (grey) and class (yellow) level based on visually distinct morphological characteristics. Modified after Rossbach et al. (2021) [15].

ID	Taxa
Polychaeta 1	*Amphiglena mediterranea*
Polychaeta 2	*Bathyvermilia* sp.
Polychaeta 3	*Bispira viola*
Polychaeta 4	*Brachiomma* sp.
Polychaeta 5	*Hydroides pseduouncinatus*
Polychaeta 6	*Hydroides* sp.
Polychaeta 7	*Janua* sp.
Polychaeta 8	*Josephella marenzelleri*
Polychaeta 9	*Pileolaria militaris*
Polychaeta 10	*Pileolaria* sp.
Polychaeta 11	Polychaeta sp. I
Polychaeta 12	Polychaeta sp. II
Polychaeta 13	Sabellida sp.
Polychaeta 14	Serpulidae sp.
Polychaeta 15	*Serpulum concharum*
Polychaeta 16	*Spirobranchus lamarckii*
Polychaeta 17	*Spirobranchus polytrema*
Polychaeta 18	*Spirobranchus triqueter*
Polychaeta 19	*Spirobranchus* sp.
Polychaeta 20	*Spororbis* sp.
Polychaeta 21	*Vermiliopsis infundibulum*
Polychaeta 22	*Vermiliopsis labiate*
Polychaeta 23	*Vermiliopsis striaticeps*
Polychaeta 24	*Vermiliopsis* sp.

SM 9 – Non-metric multidimensional scaling plot for factor 'sampling site'

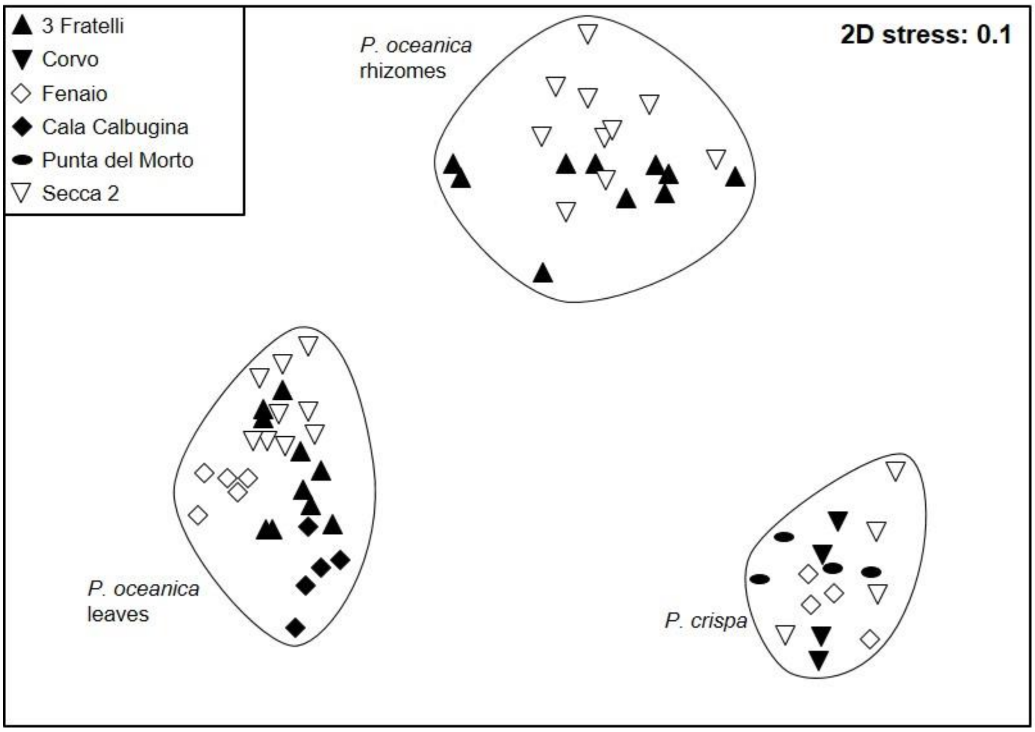

Figure S 3: Non-metric multidimensional scaling (nMDS) plot based on Bray-Curtis similarities showing that sampling sites do not influence observed biodiversity clusters.

SM 10 – Herons Formula to determine surface area enlargement factor

Supplementary Method 1:

Concept of the triangular partial surfaces (red) inside the quantification frame and the use of Herons' formula to calculate the total mean surface area of the square. a-c: sides of the respective triangle. A1-A4: respective triangle partial surfaces. Adapted from Klain (2004) [16].

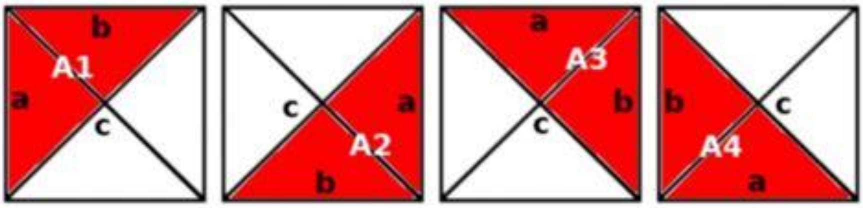

Heron´s formula:

$$A_{(triangle)} = \sqrt{s * (s-a) * (s-b) * (s-c)} \qquad s = \frac{(a+b+c)}{2}$$

$$A_{(total)} = \frac{A1 + A2 + A3 + A4}{2}$$

SM 11 – Schematic figure of the utilised Gypsum Clod Card setup

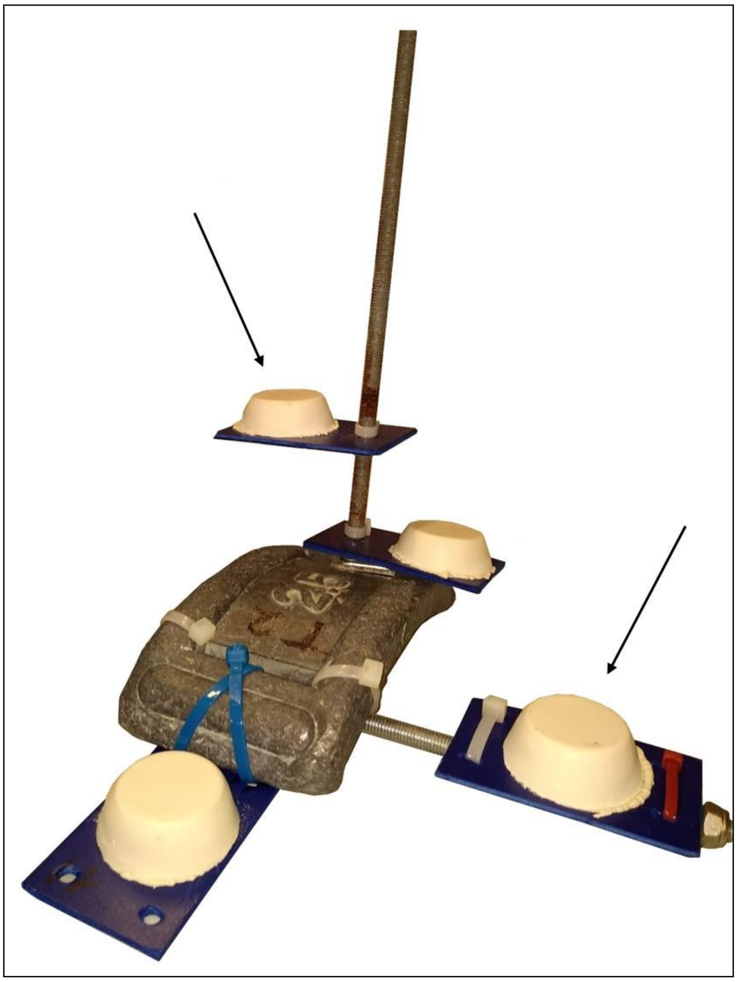

Figure S 4: Schematic figure of utilised gypsum clod card setup; data acquired for the present study based on indicated gypsum clod cards. For further information on water movement based on this setup, we refer to Schmidt et al. (2021) [17].

Supplementary References

1. Schlitzer, R. Ocean Data View. (2016).
2. Vasapollo, C. Spatio-temporal variability of plant features and motile invertebrates in Posidonia oceanica seagrass meadows. 234 (2010).
3. Riedl, R. *Fauna und Flora des Mittelmeeres*. (Seifert-Verlag, 2011).
4. Stresemann, E. *Wirbellose*. (Spektrum-Verlag, 1992).
5. Milker, Y. & Schmiedl, G. A taxonomic guide to modern benthic shelf foraminifera of the western Mediterranean sea. *Palaeontol. Electron.* **15**, (2012).
6. Murray, J. W. *British nearshore foraminiferids*. (Linnean Society of London and the Estuarine and Brackish-water Sciences Association, 1979).
7. Cimerman, F. & Langer, M. R. *Mediterranean Foraminifera*. (1991).
8. Holbourn, A., Henderson, A. S. & McLeod, N. *Atlas of Benthic Foraminifera*. (Natural History Museum, 2013).
9. Zabala, M. & Maluquer, P. *Illustrated keys for the classification of Mediterranean Bryozoa*. *Treballs - Museu de Zoologia (Barcelona)* vol. 4 (1988).
10. Hayward, P. J. & Ryland, J. S. *Marine Fauna of North-West Europe*. (Oxford University Press, 1999).
11. Bianchi, C. *Guide per il riconoscimento delle specie animali delle acque lagunari e costiere italiane*. (1981).
12. Ten Hove, H. A. & Kupriyanova, E. K. *Taxonomy of serpulidae (annelida, polychaeta): The state of affairs*. *Zootaxa* (2009). doi:10.11646/zootaxa.2036.1.1.
13. Zibrowius, H. Etude mophologique, systématique et écologique des Serpulidae (Annelida Polychaeta) de la région de Marseille. *Rec. Trav. St. Mar. Endoume Bull.* **43**, 81–252 (1968).
14. Zibrowius, H. Mise au point sur les espèces méditerranéennes de Serpulidae (Annelida Polychaeta) décrites par Stefano delle Chiaje (1822-1829, 1841-1844) et Oronzio Gabriele Costa (1861). *Tethys* **4**, 113–126 (1972).
15. Rossbach, F. I., Casoli, E., Beck, M. & Wild, C. Mediterranean red macro algae mats as habitat for high abun- dances of serpulid polychaetes. **40**, 1–13 (2021).
16. Klain, D. A. An Intuitive Derivation of Heron's Formula. *Am. Math. Mon.* **111**, 709–712 (2004).
17. Schmidt, N., El-Khaled, Y. C., Rossbach, F. I. & Wild, C. Fleshy red algae mats influence their environment in the Mediterranean Sea. *Front. Mar. Sci.* (2021) doi:10.3389/fmars.2021.721626.
