# Supplementary material for: Fleshy red algae mats act as temporary reservoirs for sessile invertebrate biodiversity
Source: Commun Biol. 2022 Jun 13;5:579. doi: 10.1038/s42003-022-03523-5 (PMC9192683; doi:10.1038/s42003-022-03523-5)
Supplement: Supplementary file 3 — Reporting Summary [file 42003_2022_3523_MOESM3_ESM.pdf]

nature research

Corresponding author(s): Yusuf C. El-Khaled

Last updated by author(s): Apr 7, 2022

# Reporting Summary

Nature Research wishes to improve the reproducibility of the work that we publish. This form provides structure for consistency and transparency in reporting. For further information on Nature Research policies, see our Editorial Policies and the Editorial Policy Checklist.

## Statistics

For all statistical analyses, confirm that the following items are present in the figure legend, table legend, main text, or Methods section.

| n/a | Confirmed | |
|---|---|---|
| ☐ | ☒ | The exact sample size ($n$) for each experimental group/condition, given as a discrete number and unit of measurement |
| ☐ | ☒ | A statement on whether measurements were taken from distinct samples or whether the same sample was measured repeatedly |
| ☐ | ☒ | The statistical test(s) used AND whether they are one- or two-sided<br>*Only common tests should be described solely by name; describe more complex techniques in the Methods section.* |
| ☐ | ☒ | A description of all covariates tested |
| ☐ | ☒ | A description of any assumptions or corrections, such as tests of normality and adjustment for multiple comparisons |
| ☐ | ☒ | A full description of the statistical parameters including central tendency (e.g. means) or other basic estimates (e.g. regression coefficient) AND variation (e.g. standard deviation) or associated estimates of uncertainty (e.g. confidence intervals) |
| ☐ | ☒ | For null hypothesis testing, the test statistic (e.g. $F$, $t$, $r$) with confidence intervals, effect sizes, degrees of freedom and $P$ value noted<br>*Give P values as exact values whenever suitable.* |
| ☐ | ☒ | For Bayesian analysis, information on the choice of priors and Markov chain Monte Carlo settings |
| ☐ | ☒ | For hierarchical and complex designs, identification of the appropriate level for tests and full reporting of outcomes |
| ☐ | ☒ | Estimates of effect sizes (e.g. Cohen's $d$, Pearson's $r$), indicating how they were calculated |

*Our web collection on statistics for biologists contains articles on many of the points above.*

## Software and code

Policy information about availability of computer code

| | |
|---|---|
| Data collection | All information is included in the manuscript. Data was collected via SCUBA diving, using standard material and loggers. |
| Data analysis | Data analysis was performed with R.Studio and Primer-E. Codes are available, see Data Availability Statement. |

For manuscripts utilizing custom algorithms or software that are central to the research but not yet described in published literature, software must be made available to editors and reviewers. We strongly encourage code deposition in a community repository (e.g. GitHub). See the Nature Research guidelines for submitting code & software for further information.

## Data

Policy information about availability of data

All manuscripts must include a data availability statement. This statement should provide the following information, where applicable:

- Accession codes, unique identifiers, or web links for publicly available datasets
- A list of figures that have associated raw data
- A description of any restrictions on data availability

All data are freely available from the corresponding author and accessible via El-Khaled et al. (2021) 52.

All code is available from the corresponding author and accessible via Daraghmeh & El-Khaled (2021) 93.

# Field-specific reporting

Please select the one below that is the best fit for your research. If you are not sure, read the appropriate sections before making your selection.

☐ Life sciences ☐ Behavioural & social sciences ☒ Ecological, evolutionary & environmental sciences

For a reference copy of the document with all sections, see nature.com/documents/nr-reporting-summary-flat.pdf

# Ecological, evolutionary & environmental sciences study design

All studies must disclose on these points even when the disclosure is negative.

| | |
|---|---|
| Study description | Recently, persistent, mat-forming fleshy red algae, previously described for the Black Sea and several Atlantic locations, have also been observed in the Mediterranean. These several centimetres high mats may displace seagrass meadows and invertebrate communities, potentially causing substantial losses of associated biodiversity. We here show that the sessile invertebrate biodiversity in these red algae mats is high and exceeds that of neighbouring seagrass meadows. |
| Research sample | All data were generated between May and July 2019 along the north-eastern and north-western coasts of Giglio Island, within the Tuscan Archipelago National Park, Tyrrhenian Sea, Italy (Supplementary Fig. S2), by SCUBA diving. |
| Sampling strategy | Via Scuba diving |
| Data collection | All data were generated between May and July 2019 along the north-eastern and north-western coasts of Giglio Island, within the Tuscan Archipelago National Park, Tyrrhenian Sea, Italy (Supplementary Fig. S2), by SCUBA diving. Data was collected by the authors as well as Alice G. Bianchi, Beltrán Montt, Mischa Schwarzmeier and Dr. Susann Roßbach. |
| Timing and spatial scale | May - July 2019, Giglio Island |
| Data exclusions | No data exclusion |
| Reproducibility | No attempts to repeat the experiment were performed. However, sampling as well as analysis are common and previously used methods, thus, the reproducibility is ensured. |
| Randomization | Samples for the present study were taken using a sampling frame (30 x 30 cm) that was randomly placed in the target area four times (i.e., each time 50 cm apart), and all sample material within the frame was carefully removed using a spatula and subsequently placed into 1 L PP-bottles (each holding a ratio of sample:water = 1:3). |
| Blinding | No blinding |

Did the study involve field work? ☒ Yes ☐ No

## Field work, collection and transport

| | |
|---|---|
| Field conditions | Smooth conditions, no current, no rainfall, temperatures >20°C. |
| Location | Giglio Island, Italy |
| Access & import/export | Samples were taken via Scuba diving. Spots were approached using a vessel belonging to the Institute of Marine Biology, Campese, Giglio Island, Italy. All samples were collected under the license for sample collection belonging to the Institute of Marine Biology, Campese. |
| Disturbance | No disturbance |

# Reporting for specific materials, systems and methods

We require information from authors about some types of materials, experimental systems and methods used in many studies. Here, indicate whether each material, system or method listed is relevant to your study. If you are not sure if a list item applies to your research, read the appropriate section before selecting a response.

## Materials & experimental systems

| n/a | Involved in the study | |
|---|---|---|
| ☒ | ☐ | Antibodies |
| ☒ | ☐ | Eukaryotic cell lines |
| ☒ | ☐ | Palaeontology and archaeology |
| ☐ | ☒ | Animals and other organisms |
| ☒ | ☐ | Human research participants |
| ☒ | ☐ | Clinical data |
| ☒ | ☐ | Dual use research of concern |

## Methods

| n/a | Involved in the study | |
|---|---|---|
| ☒ | ☐ | ChIP-seq |
| ☒ | ☐ | Flow cytometry |
| ☒ | ☐ | MRI-based neuroimaging |

# Animals and other organisms

Policy information about studies involving animals; ARRIVE guidelines recommended for reporting animal research

| | |
|---|---|
| Laboratory animals | - |
| Wild animals | Sessile invertebrates of the Mediterranean |
| Field-collected samples | Samples for biodiversity assessments were taken at six sites (two each for P. crispa mats of > 5 cm thickness and P. oceanica, and two for co-occurring habitats, resulting in four sampling sites for P. crispa and P. oceanica each, see Supplementary Fig. S2) according to accessibility and occurrence of target habitats at water depths between 28 and 30 m. |
| Ethics oversight | No ethical approval or guidance was required, all invertebrates were released to the field afterwards. |

Note that full information on the approval of the study protocol must also be provided in the manuscript.
